# Hsp90 depletion goes wild

**DOI:** 10.1186/1741-7007-10-14

**Published:** 2012-02-27

**Authors:** Mark L Siegal, Joanna Masel

**Affiliations:** 1 Center for Genomics and Systems Biology, Department of Biology, New York University, 12 Waverly Place, New York, NY 10003, USA; 2 Department of Ecology and Evolutionary Biology, University of Arizona, 1041 E. Lowell St, Tucson, AZ 85721, USA

## Abstract

Hsp90 reveals phenotypic variation in the laboratory, but is Hsp90 depletion important in the wild? Recent work from Chen and Wagner in *BMC Evolutionary Biology *has discovered a naturally occurring *Drosophila *allele that downregulates Hsp90, creating sensitivity to cryptic genetic variation. Laboratory studies suggest that the exact magnitude of Hsp90 downregulation is important. Extreme Hsp90 depletion might reactivate transposable elements and/or induce aneuploidy, in addition to revealing cryptic genetic variation.

See research article http://wwww.biomedcentral.com/1471-2148/12/25

## Commentary

Nearly 15 years ago, Rutherford and Lindquist [1] showed that reducing levels of the heat shock protein 90 (Hsp90) chaperone exposes a wide variety of phenotypes. Repeating the experiment in different strains of flies, each of which has a different genetic background, led to different Hsp90-dependent phenotypes. They argued that Hsp90 is an evolutionary 'capacitor' that creates robustness to the effects of mutations, allowing genetic variation to accumulate in a cryptic form, to be released later. This work prompted an explosion of interest in Waddington's much earlier work on the role of cryptic genetic variation in evolution [2]. Perhaps the time, technology and tractable model system had finally arrived to make progress.

In a narrow interpretation of these data, Hsp90 is seen as a contributor to the extraordinary resistance of biological systems to common perturbations such as mutation. In a more expansive view, Hsp90 perturbation is seen to promote evolvability. In a new and stressful environment, cellular demand for Hsp90 will increase beyond its supply, mimicking the effects of artificial depletion. New and stressful environments are those in which new variants, such as those revealed by Hs90 depletion, are most likely to be adaptive. This adaptation might be temporary, lasting only as long as the environmental stress. In this case, the advantage of Hsp90-dependent phenotypes might be their easily reversible nature: when the stress is over, Hsp90 levels return to normal. Alternatively, if the new environment is sustained, an Hsp90-mediated phenotype might lose its initial dependence on Hsp90 through a process of genetic assimilation. Such genetic assimilation occurs readily in the laboratory [1], and might contribute to adaptation and so increase evolvability.

There were both critics of and enthusiasts for this view of Hsp90 as evolutionary capacitor. The critics appreciated the 'coolness' of the Hsp90 system, but harbored doubts about its relevance to evolution in the wild. The enthusiasts accepted the idea of capacitance, and pressed on with investigating its mechanistic details. Recent papers have shown that a single factor is central to understanding both ecological relevance and biochemical mechanism. That factor is dosage: by exactly how much is Hsp90 activity reduced?

A recent paper in *BMC Evolutionary Biology *by Chen and Wagner [3] describes a polymorphism in the *Drosophila melanogaster *Hsp90 promoter, where a transposable element insertion halved Hsp90 expression and hence activity. When flies of the rarer low-Hsp90 type are subjected to inbreeding, their fitness declines more rapidly than that of flies that have the common Hsp90 allele [3]. This difference becomes more pronounced at higher temperature. The inference is that low Hsp90 levels increase sensitivity to mutational perturbations. One other natural polymorphism in the Hsp90 coding sequence had been previously reported [4], and this allele probably also reduces Hsp90 activity, albeit by an unknown amount. This Hsp90 variant was found to release cryptic genetic variation for normally invariant bristle traits, although not for naturally variable ones [4]. These two studies have made it clear that reductions in Hsp90 activity occur and are sometimes tolerated in the wild.

The extent to which reductions in Hsp90 activity might be tolerated, as indicated by the reduced activity of alternative natural alleles, is crucial because recent studies have demonstrated distinct thresholds of Hsp90 activity for different aspects of its biochemical function. In the original Rutherford and Lindquist [1] work, cryptic genetic variation was revealed in *Hsp90 *heterozygotes - that is, when the Hsp90 dosage was approximately halved. It is therefore plausible that revealed variation is the cause of the more rapid rate of fitness decline in the low-Hsp90 lines of Chen and Wagner [3]. However, doubt was recently cast on the capacitance hypothesis when Specchia *et al*. [5] found that reductions in Hsp90 activity interfere with the Piwi-interacting RNA (piRNA) pathway, which suppresses transposable element activity in the germline. Indeed, a morphological anomaly of the kind seen by Rutherford and Lindquist [1] was traced to a new, transposon-induced mutation, rather than to a pre-existing, cryptic allele [5].

Do the results of Specchia *et al*. [5] demand a drastic reinterpretation of over a decade of work on Hsp90-suppressed variation? Probably not. The reason is that transposon mobilization appears to require a much larger reduction in Hsp90 activity than does the revelation of cryptic variation. Moreover, Hsp90-dependent Piwi function appears not to be limited to transposition suppression in the germline, but also includes epigenetic silencing of gene expression [6]. In a particular sensitized genetic background, female *Drosophila *that were heterozygous for *Hsp90*, *piwi *or *Hop *(which encodes a protein that interacts with Hsp90 and Piwi) gave rise to a substantially increased fraction of progeny with an eye-outgrowth phenotype [6,7]. Importantly, *piwi*-heterozygous mothers do not show any elevated transposon activity [6]. Thus, it appears that Piwi (and by extension Hsp90) is haploinsufficient for suppressing cryptic variation but haplosufficient for suppressing transposon mobilization. That is, the available data suggest that halving Hsp90 activity - as in the Rutherford and Lindquist [1] experiments and in homozygotes of the natural *Hsp90 *allele identified by Chen and Wagner [3] - reveals cryptic variation but does not induce new mutations. Moreover, the greater fitness decline seen by Chen and Wagner [3] upon inbreeding their low-Hsp90 lines is almost certainly caused by revealed pre-existing variation rather than by correlation with a high transposon load, because the low-Hsp90 genetic background (and therefore any new transposon insertions it contained) and the wild-type genetic background were mixed evenly by crossing them several times before each was inbred.

Parallel to the work in *Drosophila*, recent work in the yeast *Saccharomyces cerevisiae *has demonstrated roles for Hsp90 in revealing cryptic variation and in maintaining genome integrity. Jarosz and Lindquist [8] identified a large reservoir of Hsp90-dependent genetic variation in yeast populations and mapped several pre-existing alleles that cause new phenotypes upon Hsp90 impairment. Chen *et al*. [9] found that Hsp90 impairment induces yeast chromosome instability, leading to aneuploidy. Again, it appears that Hsp90 must be reduced to a greater extent to cause aneuploidy than to cause the release of cryptic variation [9]. Still, the production of aneuploidies by Hsp90 impairment remains an intriguing new mechanism of Hsp90-modulated diversity, especially because: 1) some aneuploidies appear to be beneficial under stress; and 2) a common form of aneuploidy, monosomy, is reversible by chromosome duplication [9]. Under severe stress, Hsp90-related aneuploidy might therefore provide the same kind of transient benefit in yeast that can be provided by capacitance.

**Figure 1:**
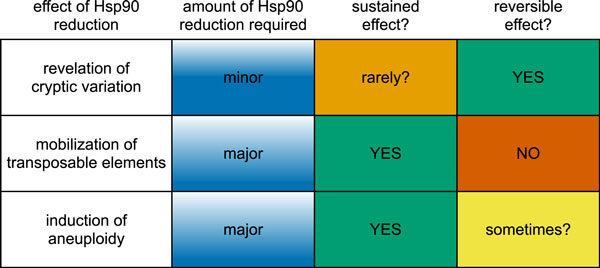
**The extent of Hsp90 depletion affects the mechanism by which phenotypic effects are induced**. To work as an evolutionary capacitor, facilitating evolvability above and beyond the effects of mutagenesis, induced variation must persist for multiple generations, but also be reversible once genetic assimilation is complete. None of the three proposed effects of Hsp90 depletion clearly meets both hurdles.

For capacitance to provide evolvability that goes above and beyond the effects of induced mutagenesis, the effects of Hsp90 depletion must: 1) persist for enough generations to allow genetic assimilation of the adaptive phenotype to take place; and 2) be reversible once this process is complete [2]. None of the proposed mechanisms of Hsp90 depletion unambiguously meet both criteria (Figure 1). Persistence is unknown for revelation of cryptic genetic variation, transposable element mobilization is irreversible, and aneuploidy is reversible only some of the time. An additional important criterion is that Hsp90-induced phenotypes are at least occasionally adaptive rather than 'monstrous' [2].

Research on Hsp90 in the past two years has shown that the modulation of phenotypic variation might have many mechanisms and that natural genetic variation can mimic the effects on phenotypic variation seen in laboratory experiments. An important but still unanswered question, however, is the degree to which Hsp90 is special. In other words, if similar depletion experiments were performed on each of the other genes in the genome, would Hsp90 stand out? There are some indications that it might not. In a screen for destabilization of yeast morphology to environmental noise, genes affecting chromosome organization were overrepresented, but chaperones were not [10]. Although the phenotypes revealed by *Drosophila *Hsp90 depletion in the laboratory are exceptionally diverse, many of them are monstrous and clearly irrelevant to adaptation. In another 15 years, will research interest in Hsp90 as a model capacitor be viewed as a historical accident? Not only does yeast contain a large number of other candidate capacitor genes [10], but a recent yeast study traces extensive natural variation to the presence of epigenetically inherited prions, and this variation is demonstrably adaptive in some environments and genetic backgrounds [11]. Hsp90 is not the only contender for attention. Evolutionary capacitance may be everywhere, and the search for it has only just begun.
